# The protective effect of human renal sinus fat on glomerular cells is reversed by the hepatokine fetuin-A

**DOI:** 10.1038/s41598-017-02210-4

**Published:** 2017-05-23

**Authors:** R. Wagner, J. Machann, M. Guthoff, P. P. Nawroth, S. Nadalin, M. A. Saleem, N. Heyne, A. Königsrainer, F. Fend, F. Schick, A. Fritsche, N. Stefan, H.-U. Häring, E. Schleicher, D. I. Siegel-Axel

**Affiliations:** 1 Division of Endocrinology, Diabetology, Angiology, Nephrology, and Clinical Chemistry, Department of Internal Medicine IV, University Hospital Tübingen, Tübingen, Germany; 2 Institute for Diabetes Research and Metabolic Diseases of the Helmholtz Center Munich at the Eberhard Karls University Tübingen, Tübingen, Germany; 3 German Center for Diabetes Research (DZD), Neuherberg, Germany; 4 Department of Diagnostic and Interventional Radiology, Section on Experimental Radiology, University Hospital Tübingen, Tübingen, Germany; 5 Department Medicine I and Clinical Chemistry, University of Heidelberg, Heidelberg, Germany; 6 Department of General, Visceral and Transplant Surgery, University of Tübingen, Comprehensive Cancer Center, Tübingen, Germany; 7 Bristol Renal and Children’s Renal Unit, School of Clinical Sciences, University of Bristol, Bristol, UK; 8 Institute of Pathology and Neuropathology, University of Tübingen, Tübingen, Germany

## Abstract

Renal sinus fat (RSF) is a perivascular fat compartment located around renal arteries. In this *in vitro* and *in vivo* study we hypothesized that the hepatokine fetuin-A may impair renal function in non alcoholic fatty liver disease (NAFLD) by altering inflammatory signalling in RSF. To study effects of the crosstalk between fetuin-A, RSF and kidney, human renal sinus fat cells (RSFC) were isolated and cocultured with human endothelial cells (EC) or podocytes (PO). RSFC caused downregulation of proinflammatory and upregulation of regenerative factors in cocultured EC and PO, indicating a protective influence of RFSC. However, fetuin-A inverted these benign effects of RSFC from an anti- to a proinflammatory status. RSF was quantified by magnetic resonance imaging and liver fat content by ^1^H-MR spectroscopy in 449 individuals at risk for type 2 diabetes. Impaired renal function was determined via urinary albumin/creatinine-ratio (uACR). RSF did not correlate with uACR in subjects without NAFLD (n = 212, p = 0.94), but correlated positively in subjects with NAFLD (n = 105, p = 0.0005). Estimated glomerular filtration rate ﻿(eGRF) was inversely correlated with RSF, suggesting lower eGFR for subjects with higher RSF (r = 0.24, p < 0.0001). In conclusion, our data suggest that in the presence of NAFLD elevated fetuin-A levels may impair renal function by RSF-induced proinflammatory signalling in glomerular cells.

## Introduction

Obesity and particularly disproportionate fat distribution is considered to be not only involved in the development of type 2 diabetes (T2D) and cardiovascular diseases, but also in the progression of chronic kidney disease^1–3^. The focus of interest in the last years is to study the exclusive role of perivascular adipose tissue (PVAT) which signals locally in a paracrine and vasocrine fashion to the vascular wall through outside-to-inside signalling. Beside visceral and subcutaneous fat depots which exert systemic effects, there are also locally acting fat depots such as PVAT. Previous studies have shown that PVAT is not only a physical support of arteries but acts as endocrine organ secreting adipokines, (pro)-inflammatory proteins and other bioactive factors^4–6^.

Recently we showed that human PVAT around arteries from different locations and in crosstalk with other organs represents a very active fat compartment with angiogenic potential^7, 8^. It expresses and releases adipokines, (pro)-inflammatory cytokines, e.g. interleukins, monocyte-chemoattractive protein (MCP-1) and specific angiogenic/regenerative factors, such as hepatocyte growth factor (HGF), which all may contribute to vascular diseases^7^. Since PVAT is in close contact with vascular cells of larger arteries as well as vasa vasorum pervading the adventitia without any barrier, we examined the interactions of human perivascular fat cells (PVFC) with human arterial endothelial cells (EC). We found that regenerative factors, predominantly HGF, but not (pro)-inflammatory factors, were upregulated in EC by the crosstalk with PVFC and vice versa, indicating the higher angiogenic potential of this fat cell type^7^.

Other clinical studies of our group showed that the fatty liver is associated with a dysregulated secretion of specific cytokines (hepatokines)^9^ with signaling properties for other tissues^10, 11^. Among them, fetuin-A induces insulin resistance and, in concert with fatty acids, subclinical inflammation^12^. Since there is also a link between the fatty liver and a cardiovascular risk^13^, we have simulated the crosstalk of pathophysiological plasma fetuin-A levels secreted by the fatty liver with (peri)vascular “organs”. We found a significant downregulation of HGF but an upregulation of proinflammatory factors under the influence of high fetuin-A levels^14^.

Another independent perivascular fat compartment located around the renal hilum, the renal sinus fat (RSF), was recently identified^15, 16^. We could demonstrate that under exercise conditions an increased RSF mass - quantified by magnetic resonance imaging (MRI) - was associated with elevated microalbuminuria^17^ indicating that this fat depot may be involved in the development and aggravation of renal lesions^18^. In a recently published cross-sectional study using computed tomography, asymmetrical deposition of adipose tissue into the renal sinus was described^19^. This was found to be related with the visceral adipose tissue amount but reductions in visceral adipose tissue volume were not accompanied by reductions in renal sinus fat accumulation. As emphasized in a very recent commentary, an increasing number of studies are providing proof that renal sinus fat plays an important role in obesity-induced renal injury^20^ which could be diagnosed and linked with early biomarkers of kidney injury^19^.

However, a very limited number of functional *ex-vivo* studies with renal sinus fat cells (RSFC) are published until now. In the present combined *in vitro* and *in vivo* study we were prompted to answer the question if human RSFC of this very specific fat compartment differs from other PVFC which we described earlier, as well as from visceral and subcutaneous fat cells of human donors. Furthermore, aim of this study was to examine if factors released from RSFC may affect the function of EC and podocytes (PO) in renal glomerula. According to our findings in PVFC, the impact of fetuin-A on glomerular cells was investigated in the same experimental setting because the fatty liver may also negatively affect renal function in humans^21^. To prove a potential impact of our *in vitro* findings for prediabetic subjects, a relationship of RSF volume on kidney integrity was analyzed separately for individuals with and without non alcoholic fatty liver disease (NAFLD).

## Results

### Characteristic features of isolated primary human perivascular and renal sinus fat cells

**Figure 1:**
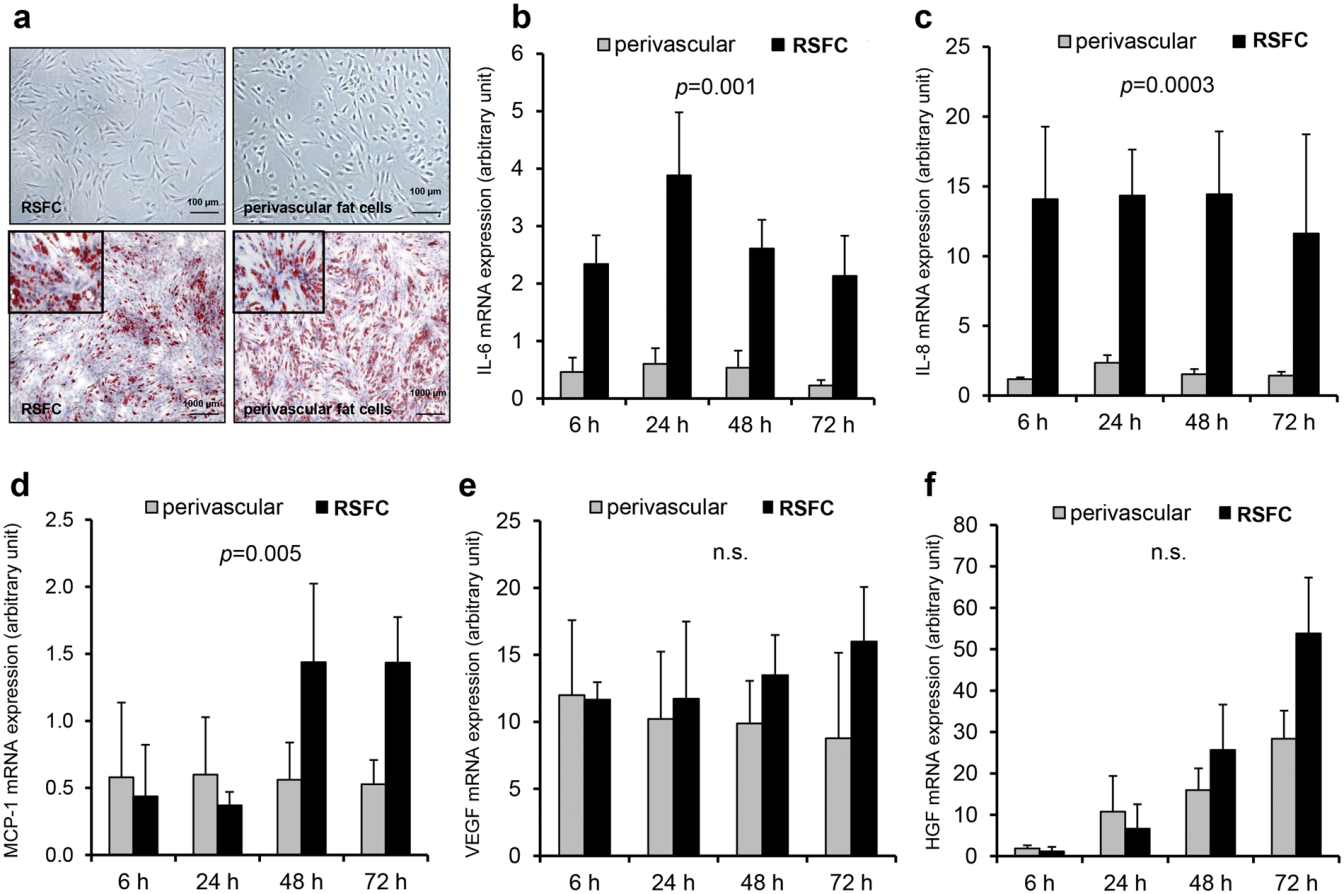
(**a**) Growth pattern and characterization of RSFC and PVFC after isolation. Upper panel: characteristic growth of RSFC (left) and PVFC (right) in culture. Lower panel: Oil red stained differentiated adipocytes show typical cytoplasmic lipid droplets (magnification ×4, as detail in the inset ×40) (**b–f**) Comparison of the time dependent mRNA-expression of different (pro)-inflammatory and regenerative factors in PVFC (grey bars) versus RSFC (black bars) determined by real-time PCR (light cycler) after 6, 24, 48 and 72 h of cultivation. (**b**) IL-6, (**c**) IL-8, (**d**) MCP-1, (**e**) VEGF and (**f**) HGF. Statistical analysis: random-slopes linear mixed models with the donor and the observation time as random effects. Values are mean ± SEM from 4 independent experiments (*p*-values are shown for the basal mRNA-expression versus the housekeeping gene RPS13).

RSFC could be isolated, characterized and subcultured from RSF of four different patients. Functional properties and effects of this unique renal perivascular fat compartment were then examined *ex vivo*. Cultured RSFC showed a spindle shape with cytoplasmic protrusions and cluster formation (Fig. 1a, upper level) similar to PVFC isolated from arm arteries^7^. When exposed to differentiation medium, the cytoplasm of nearly each RSFC was densely packed with lipids and both RSFC and PVFC exhibited a high capacity to differentiate into mature adipocytes (>90% of the cells), as detected by oil red staining (Fig. 1a, lower level). FACS analyses indicated that the primary cultures were not contaminated with other cell types (Supplementary Fig. S1).

### Comparison of mRNA levels and protein production of proinflammatory and regenerative factors in human fat cells obtained from RSF and other human fat compartments

RSFC showed an increased IL-6 (5-fold) and IL-8 (>10-fold) mRNA-expression compared to PVFC during all time points studied (Fig. 1b,c), while MCP-1 mRNA levels were only slightly higher than in PVFC (Fig. 1d). VEGF mRNA-expression was similar in both fat cell types (Fig. 1e) whereas HGF was somewhat (but not significantly) higher than in RSFC (Fig. 1f).

We also compared the basal expression of these factors in visceral fat cells (VFC) and subcutaneous fat cells (SCFC) (Supplementary Fig. S2). These respective results indicate that the expression patterns of (pro)-inflammatory factors are similarly elevated in VFC and RSFC, when compared to PVFC or SCFC, whereas the regenerative factor VEGF did not differ significantly. According to our previous studies^7^ HGF is generally higher expressed in PVFC compared to SCFC or VFC, but this level was even exceeded in RSFC after 72 h (Fig. 1f and Supplementary Fig. S2e).

In RSFC a remarkably high production of IL-6, IL-8, MCP-1, HGF and VEGF was detectable. Very high levels of the (pro)-inflammatory proteins IL-6 (after 24 h: 390 ± 175 pg/ml, after 48 h: 585 ± 223 pg/ml and after 72 h: 679 ± 301 pg/ml, n = 6), as well as IL-8 (after 24 h: 243 ± 127 pg/ml, after 48 h: 684 ± 379 pg/ml and after 72 h: 905 ± 510 pg/ml, n = 6) were found in supernatants of RSFC which were comparable to VFC^7^. The protein levels reflected the mRNA-expression data. In addition, RSFC secreted higher levels of the chemoattractant MCP-1 (after 24 h: 110 ± 59 pg/ml, after 48 h: 214 ± 68 pg/ml and after 72 h: 352 ± 82 pg/ml, n = 6) which exceeded those of the other fat cell types described previously^7^, but within a comparable concentration range as found in PVFC.

HGF protein levels in RSFC (after 24 h: 1410 ± 513 pg/ml, after 48 h: 2864 ± 1540 pg/ml and after 72 h: 7242 ± 303 pg/ml, n = 6) were in general comparable to those found in PVFC, but were remarkably higher than in VFC and SCFC, a finding that was also observed previously^7^. VEGF proteins increased time-dependently (after 24 h: 179 ± 24 pg/ml, after 48 h: 180 ± 56 pg/ml and after 72 h: 365 ± 47 pg/ml, n = 6). These levels were also in a comparable concentration range as those from PVFC. Together, these results show that the protein production corresponded to the respective mRNA-expression.

### Effect of RSFC on mRNA-expression pattern of human EC and PO in coculture

**Figure 2:**
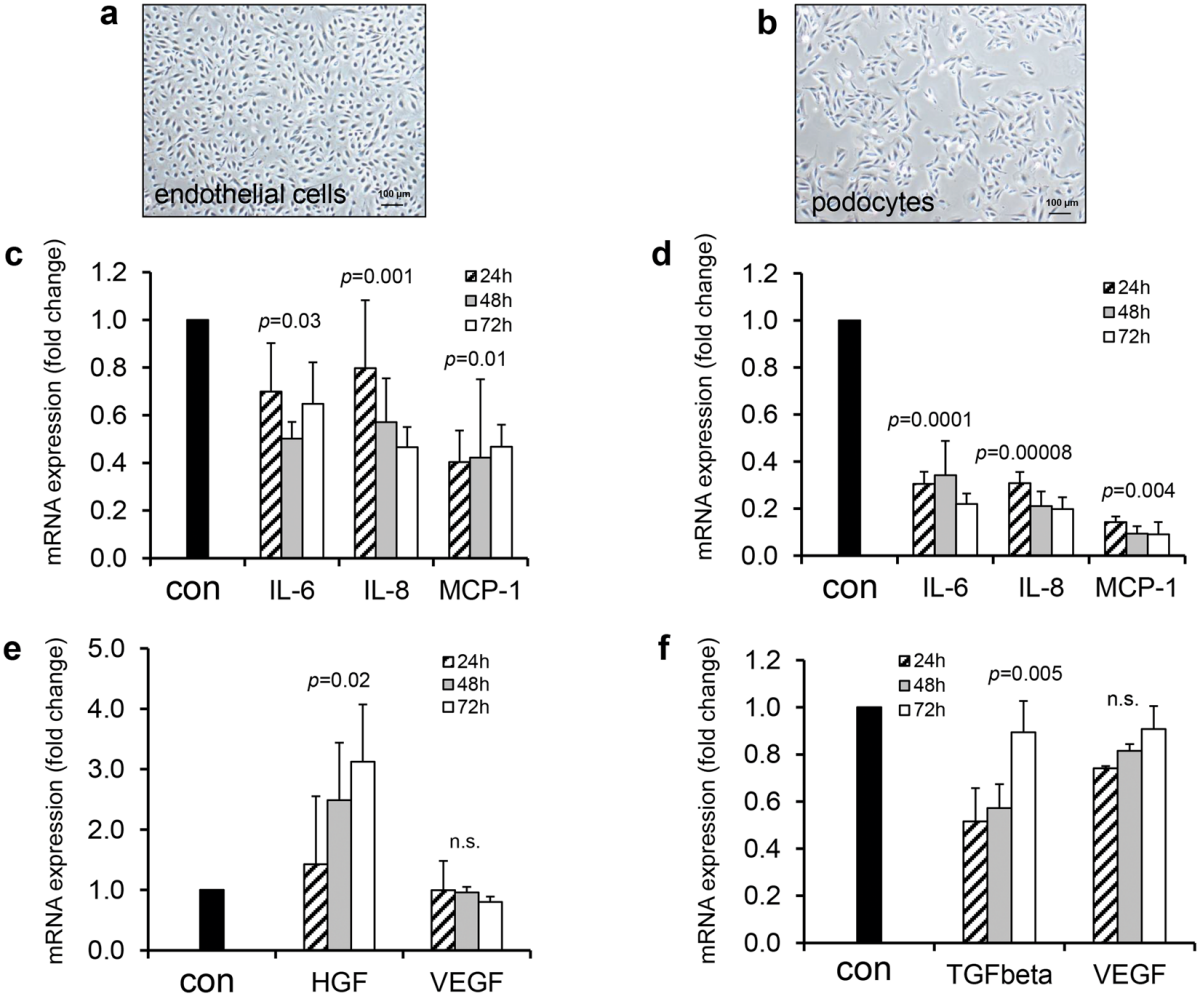
Influence of RSFC on human glomerular cells. Growth pattern of EC (**a**) and PO (**b**). mRNA-expression of (pro)-inflammatory (**c**) and regenerative factors (**e**) in EC and in PO (**d,f**), respectively. Of note, PO did not express HGF. Cells were cocultured with RSFC for 24, 48 and 72 h. Statistical analysis: random-slopes linear mixed models with the donor and the observation time as random effects, and the culture as fixed effects (mono- vs coculture) for that *p*-values are given. Values are mean ± SEM from six independent experiments.

The isolation of pure EC was possible in 80% of vessel preparations. EC exhibited the characteristic cobblestone pattern (Fig. 2a). The growth pattern of PO is shown in Fig. 2b. Monocultured EC and PO showed a remarkable mRNA expression of (pro)-inflammatory and regenerative factors during the overall cultivation period of 72 h (Fig. 2c–f). The mRNA-expression of the respective EC- and PO-monoculture served as control. PO did not express relevant HGF levels (not shown).

When EC were cocultured with RSFC, IL-6, IL-8 and MCP-1 mRNA-expression was downregulated significantly in EC (Fig. 2c), whereas HGF was upregulated significantly in between 24–72 h (Fig. 2e). VEGF did not change. ICAM-1 and predominantly ALCAM were downregulated (Supplementary Fig. S3a). Compared to EC, in PO a strong downregulation of IL-6, IL-8 and MCP-1 (Fig. 2d) and a minor reduction of VEGF mRNA-expression was observed (Fig. 2f). ICAM-1 and ALCAM were significantly downregulated (Supplementary Fig. S3b). Since no relevant mRNA-expression of the regenerative factor HGF was detectable in PO, but its antagonist TGF-β, a well-known profibrotic factor in the kidney, this factor was analyzed additionally in PO. TGF-β mRNA-expression was also significantly downregulated during coculture of PO with RSFC (Fig. 2f). All of these effects persisted over the entire cultivation period of 24–72 h.

The vice versa effects of EC on RSFC were similar to those previously described for PVFC cocultures^7^. While in RSFC cocultivated with EC or PO the mRNA-expression of (pro)-inflammatory factors was induced (Supplementary Fig. S4a and c), HGF and VEGF were only moderately affected in RSFC cocultivated with EC (Supplementary Fig. S4b). The influence of cocultured PO on (pro)-inflammatory (Supplementary Fig. S4c) and regenerative factors (Supplementary Fig. S4d) in RSFC was even more pronounced.

### The influence of fetuin-A on RSFC

**Figure 3:**
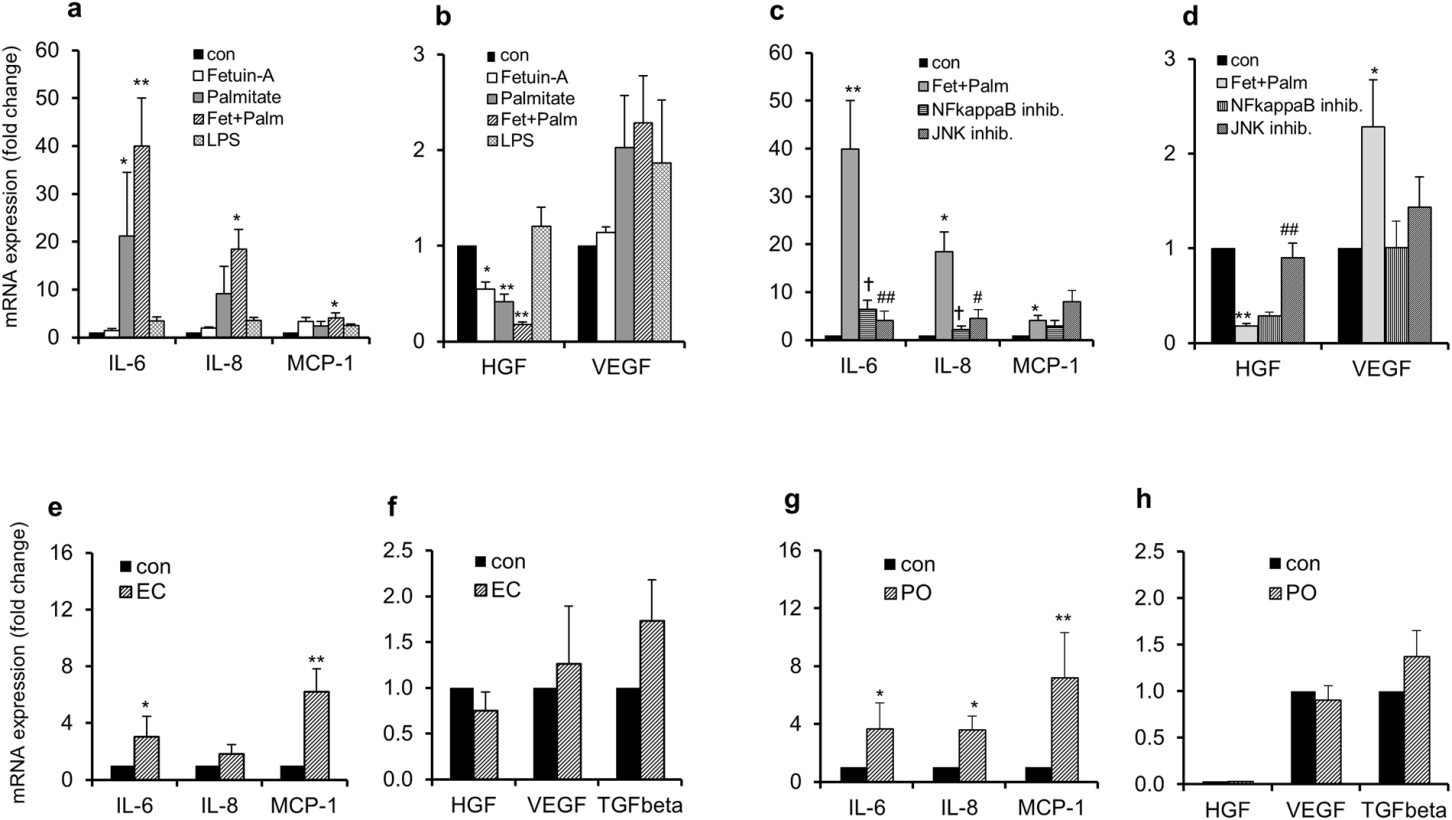
Influence of fetuin-A and/or palmitate on RSFC. Effect of 600 µg/ml fetuin-A ± 50 µmol/l palmitate, palmitate per se, and LPS (as endotoxin control) on the mRNA-expression of (**a**) the (pro)-inflammatory proteins IL-6, IL-8 and MCP-1, and (**b**) the regenerative factors HGF and VEGF 24 h after treatment of RSFC. (**c,d**) Treatment of RSFC with an NFkappaB inhibitor or JNK inhibitor. Statistical analysis: ANOVA and Tukey post hoc test. Values are mean ± SEM from three independent experiments (*p < 0.05 and **p < 0.01 for fetuin-A and/or palmitate-treated cells versus controls (con) defined as 1 as well as ^#^ *p* < 0.05 and ^##^ *p* < 0.01 or ^†^<0.05 and ^††^<0.01 for fetuin-A/palmitate-treated cultures versus identical cultures pretreated with the respective inhibitors). Effects of fetuin-A-treated RSFC on the mRNA-expression of (pro)-inflammatory and regenerative factors in cocultured EC and PO. Cocultures of RSFC with EC or PO were treated with fetuin-A (600 µg/ml) for 24 h. The mRNA-expression of (pro)-inflammatory factors (**e**,**g**) and regenerative factors (**f**,**h**) in EC and PO, respectively, is shown. Statistical analysis: paired t-test to analyze differences in measurement pairs from the same donors. Values are mean ± SEM from four independent experiments (*p < 0.05 and **p < 0.01 for cocultures treated with fetuin-A versus controls (con) defined as 1).

To mimic a potential influence of the fatty liver on RSF and, consequently, on glomerular cells, the hepatokine fetuin-A was added to the culture media of RSFC. The dose of 600 μg/ml fetuin-A is representative of plasma levels in obese individuals^21, 22^. IL-6 and lL-8 mRNA-expression in RSFC was slightly upregulated by fetuin-A or palmitate, but the combination potentiated (up to 50%) the effects of each single stimulator (Fig. 3a). The moderate stimulation by LPS indicates that the effects were not caused by relevant impurities in fetuin-A preparations. MCP-1 mRNA expression was also stimulated by fetuin-A, but not induced additionaly by palmitate to the same extent as found for the (pro)-inflammatory factors. The stimulation of VEGF was also slightly augmented by palmitate (Fig. 3b). In contrast, HGF was significantly downregulated under the influence of fetuin-A with (5-fold) or without (2-fold) palmitate (Fig. 3b). Previously, Pal *et al*.^23^ demonstrated in mice that fetuin-A is an endogenous ligand that physically interacts and allows palmitate–TLR4 receptor crosstalk and signalling, resulting in the production of (pro)-inflammatory cytokines, a finding that we could previously support with human fat cell data^14^. We demonstrated earlier in PVFC that the (pro)-inflammatory effects of fetuin-A were mediated by the NFκB pathway and blocked by TLR4 inhibition^14^. However, in this study with RSFC we tried to answer the question if also NFκB-independent protein kinases are involved downstream of TLR4 activation. Both the NFκB inhibitor and the JNK inhibitor almost completely abrogated the fetuin-A/palmitate-induced stimulatory effects on IL-6 and IL-8 (Fig. 3c). The effects on MCP-1 were just reversed slightly by the NFκB inhibitor. The MEKl/2 and p38MAPkinase inhibitors had no significant effects (data not shown). In contrast to these TLR4-mediated stimulatory effects on interleukins, HGF mRNA-expression was potently inhibited by fetuin-A/palmitate which could not be abrogated by the NFκB inhibitor (Fig. 3d), but completely abrogated by the JNK inhibitor (Fig. 3d). Finally, the stimulatory effects on the regenerative factor VEGF could be reversed by either inhibitor (Fig. 3d). The data indicate that the effects of fetuin-A on (pro)-inflammatory factors and HGF are regulated by different signaling pathways.

### Effects of fetuin-A-treated RSFC on EC and PO in cocultures

To prove that RSFC treated with fetuin-A may influence glomerular EC or PO, cocultures with RSFC were studied. Fetuin-A caused a strong upregulation of Il-6 and, particularly MCP-1 (up to 7-fold) mRNA-expression in EC, when compared to untreated control cultures (Fig. 3e). TGF-β mRNA expression increased whereas HGF decreased slightly but not statistically significant (Fig. 3f). Since TGF-β is known to induce CTGF *(*connective tissue growth factor) expression e.g. in renal mesangial cells, we studied also this matricellular and pro-fibrotic protein in RSFC. CTGF was expressed markedly by all three cell types and fetuin-A caused a slight upregulation of CTGF mRNA expression in RSFC (*p* = 0.06, control cultures: 70.22 ± 30.57 versus fetuin-A treated cultures 101.41 ± 51.74 mRNA expression related to the housekeeping RPS13, n = 4, Supplementary Fig. S5).

The mRNA data were underlined by protein analysis: IL-6, IL-8, MCP-1 and VEGF protein secretion was upregulated (Supplementary Fig. S6), HGF remained unchanged. The IL-6 and IL-8 mRNA-expression and protein secretion which were downregulated in PO by the coculture with RSFC (see Fig. 2d), were not only abolished but even upregulated considerably by fetuin-A-treated RSFC (Fig. 3g). MCP-1 and VEGF protein secretions were increased 1.5-2-fold (Fig. 3h, and Supplementary S6b).

Since the increased MCP-1 expression, predominantly in EC, indicates an enhanced attraction of mononuclear cells, we also studied the expression of the adhesion molecules ICAM-1 and ALCAM in RSFC, EC and PO under the influence of fetuin-A. We found a trend for an upregulation of ICAM-1 and ALCAM in PO and a significant stimulation of ALCAM in RSFC and EC (Supplementary Fig. S7).

### Immunohistochemical detection of macrophages and vascularization in human RSF

**Figure 4:**
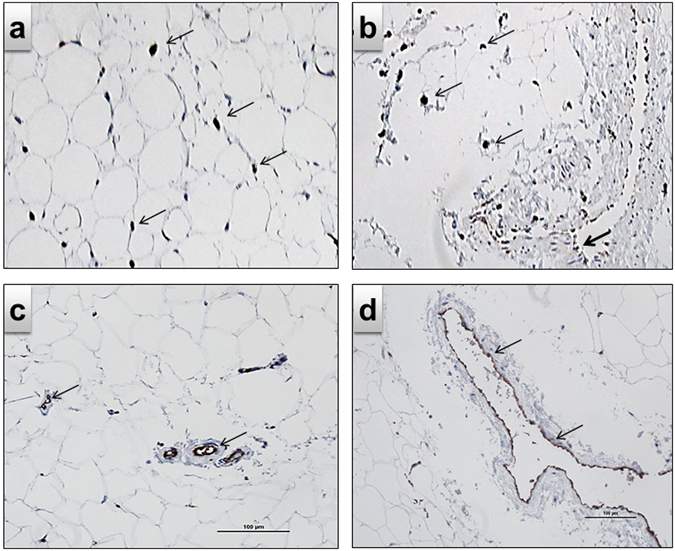
Immunohistochemical detection of macrophages and EC. RSF obtained from human renal resections: (**a**) CD 68, specific for both M1 and M2 macrophages, and (**b**) CD 206, a C-type lectin on macrophages, specific for M2 macrophages which are located inside the fat tissue (thin arrows) but also along blood vessels (bold arrow). Microvascularization detected by CD31 staining of EC (**c**) located between fat cells, and (**d**) larger vessels between the fat tissue. The cells are marked by brown staining (arrows). The scale bars represents 100 µm.

To prove the presence of macrophages in human RSF, which could have been attracted by MCP-1, and bind to ICAM-1 or ALCAM, stainings against CD68 (M1 and M2 macrophages) and CD206, a C-type lectin on M2 macrophages, were performed in human RSF resections. A remarkable number of both activated proinflammatory M1 and deactivated/(anti)-inflammatory M2 macrophages are present between the large, fat-filled adipocytes (Fig. 4a,b) and an intense micro- (Fig. 4c) and macro- (Fig. 4d) vascularization, which was detected by CD31-staining of EC.

**Figure 5:**
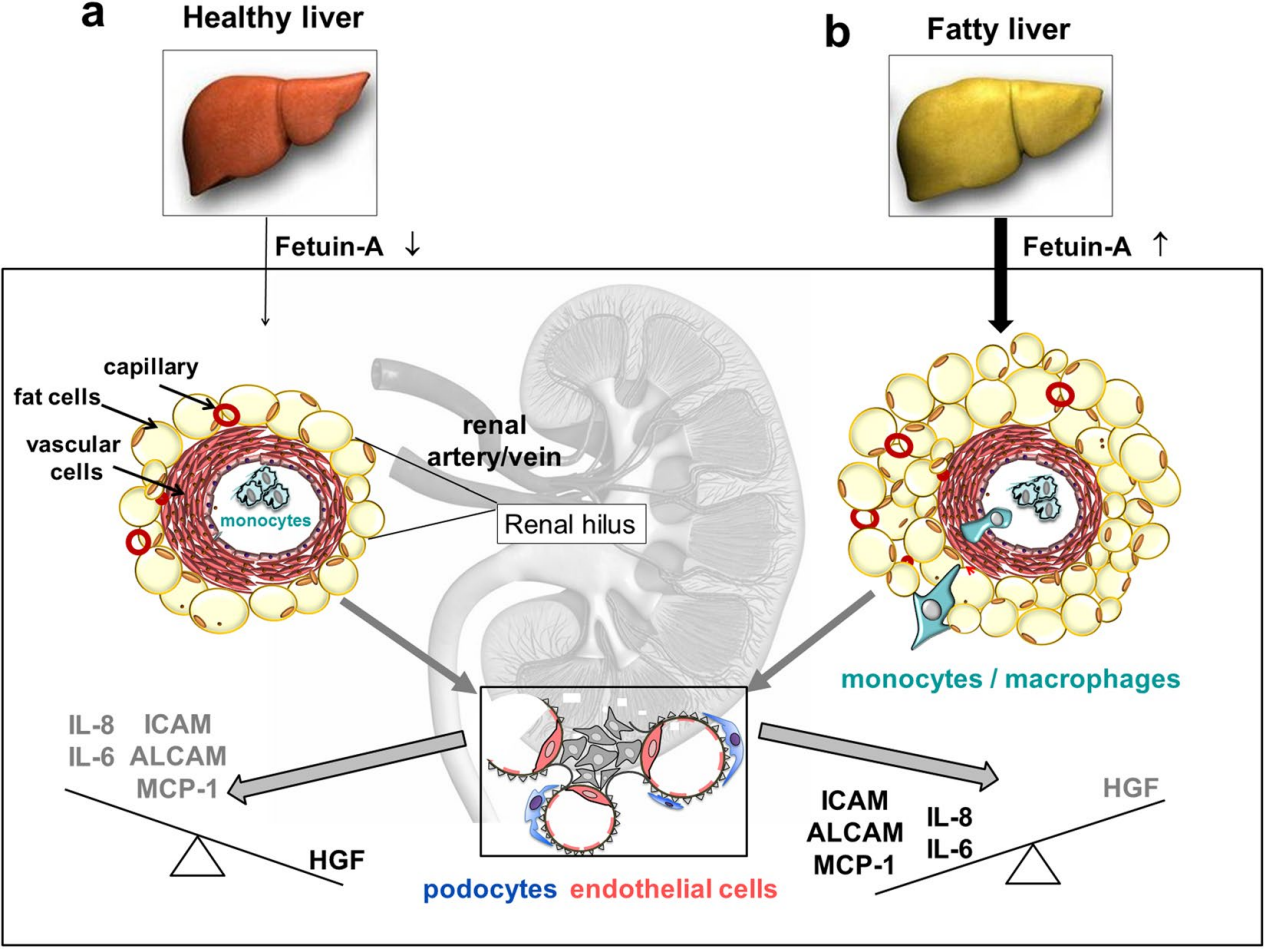
Proposed mechanism of the balance between RSFC-released (pro)-inflammatory cytokines and adhesion molecules, as well as the regenerative factor HGF (VEGF and the counterplayer TGF-β are not changed significantly and are therefore not shown for clarity). The crosstalk with the fatty liver via the increased hepatokine fetuin-A reversed the balance from a (**a**) ‘healthy’ to an (**b**) ‘unhealthy’ ((pro)-inflammatory and chemoattractive) status.

As shown in Fig. 5, we propose a mechanism of the balance between RSFC-released (pro)-inflammatory cytokine and adhesion molecules and regenerative factors. The crosstalk with the fatty liver reverses the balance to an unhealty status where monocytes/macrophages are attracted.

### Renal sinus fat in humans

**Figure 6:**
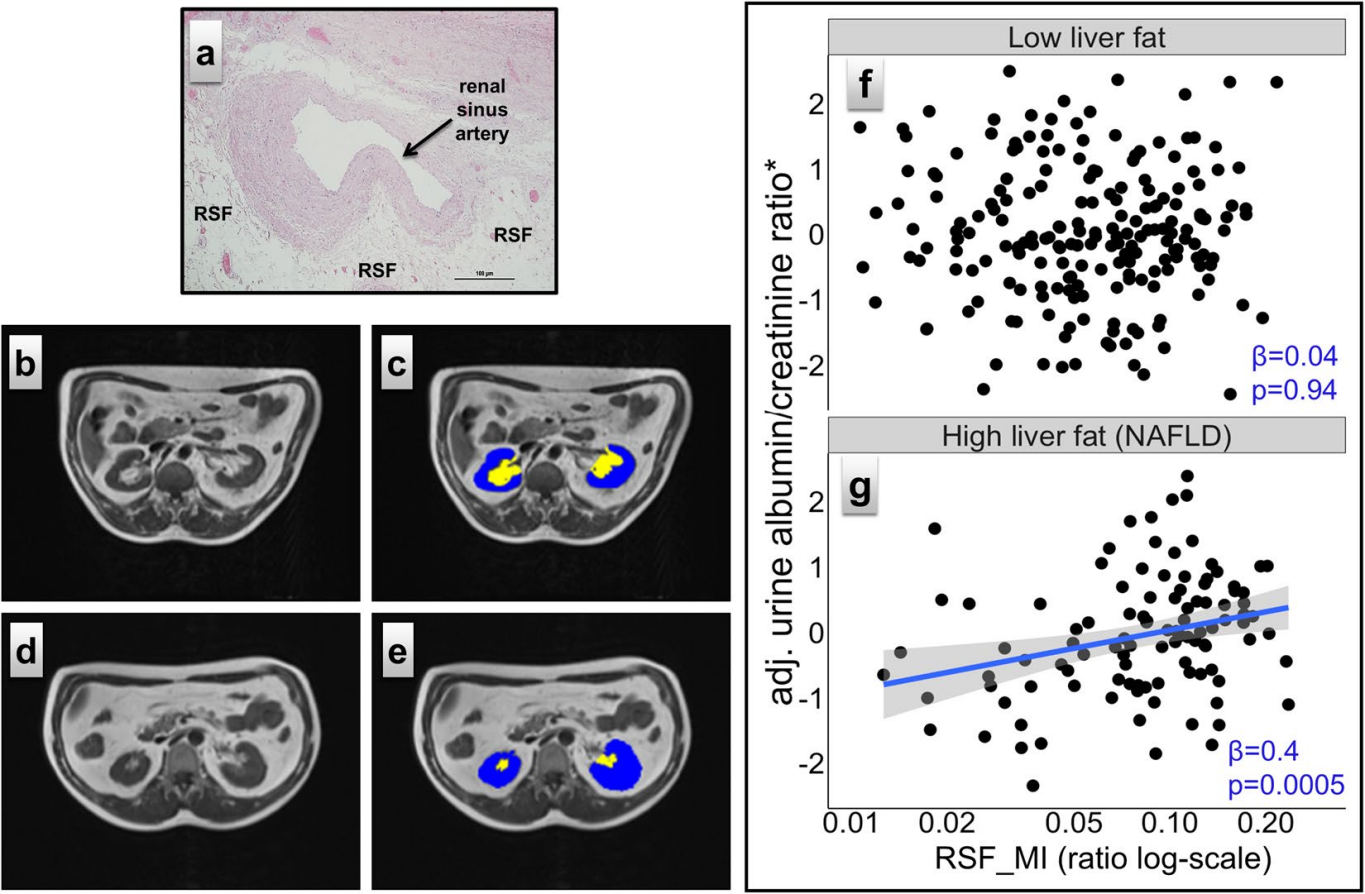
Human renal sinus fat. (**a**) The human histological section shows the close proximity of RSF to the vascular wall. (**b–e**) Transverse T1-weighted MRI slices of two 61-year-old male participants: (**b,d**) Both kidneys are shown at the level of the hili. While the participants have comparable BMI, the sizes of the RSF compartments are strikingly different. (**c,e**) The same MRI slices as on the left side, but with highlighted RSF areas in yellow and kidney areas in blue to illustrate the calculation of ${\rm{RSF}}\_{\rm{MI}}=\frac{yellow\,area}{yellow\,area+blue\,area}$. RSF_MI of the participant on Fig. 6b,c is 0.23, whereas RSF_MI of the participant on Fig. 6d,e is only 0.07. (**f,g**) Association of RSF_MI with urinary albumin-to-creatinine ratio (uACR) stratified by the liver fat content (liver fat content >5.6% was classified as fatty liver, NAFLD). RSF_MI is displayed on a log-scale. uACR was transformed to approximate normal distribution. Non-diabetic residuals adjusted for sex, age and BMI are plotted. RSF area did not correlate with uACR in subjects without fatty liver (n = 212) but positive relationships were found in subjects with fatty liver (n = 105).

A representative human histological section shows the close proximity of human RSF to the vascular wall without any barrier to the arterial adventitia (Fig. 6a). RSF area was measured in 449 participants. Anthropometric and clinical data of the studied cohort are shown in Table 1. The percentage of cross-sectional fat area in the renal sinus compared to the cross-sectional area of the kidney was separately assessed by MR imaging on both sides. Examples of two participants are shown in Fig. 6b–e. RSF_MI of the participant on Fig. 6b,c is 0.23, whereas RSF_MI of the participant on Fig. 6d,e is only 0.07. Kidneys on the left side showed significantly more fat in their renal sinus than kidneys on the right side (RSF_MI = 0.088 vs 0.054, p < 0.0001). Mean RSF_MI was higher in males than in females (0.091 vs. 0.055, p < 0.0001), and correlated positively with age (R^2^ = 0.21, beta = 0.17, p < 0.0001) and BMI (R^2^ = 0.06, beta = 0.0023, p < 0.0001).

**Table 1:** Anthropometric and clinical data of the studied cohort interquartile range.

Trait (N = 449)	Median	Interquartile range
**Age (years)**	45	33–54
**Sex (f/m)**	249/200	
**BMI (kg/m** ^**2**^ **)**	29.4	26.87–32.11
**Fasting glucose (mmol/l)**	5.22	4.89–5.56
**Post-challenge glucose (mmol/l)**	6.33	5.56–7.22
**Renal sinus fat (ratio)**	0.07	0.03–0.1
**Plasma creatinine (µmol/l)**	71	62–88
**Estimated GFR (ml/min/1.73** **m** ^**2**^ **)**	94.1	81.08–107.5
**Urine albumin-to-creatinine ratio (mg/g)**	8.5	6.04–15.49

To evaluate the relevance of *in vitro* findings the influence of RSF on urinary albumin excretion rate in non-diabetic subjects was determined. We found a trend for association of RSF area with albumin-to-creatinine ratio (uACR) (p = 0.07) after adjustment for sex, age and BMI. Our biological hypothesis suggested that a deleterious impact of RSF on renal function may be present with a concomitant NAFL. We found an interaction between RSF and liver fat content fitted on uACR (p = 0.03), showing that liver fat content could indeed modulate the association of RSF with uACR. In the stratified analysis, subjects without NAFL (liver fat content <5.6%, n = 212) RSF did not associate with uACR (beta = 0.04, p = 0.94, Fig. 6f). In subjects with NAFL (liver fat content >5.6%, n = 105) a significant association between RSF and uACR (beta = 0.40, p = 0.0005, Fig. 6g) was found. Plasma fetuin-A concentrations were associated with uACR in subjects with fatty liver (n = 41, beta = 0.3, p = 0.05), while no association was seen in subjects without fatty liver (n = 88, beta = −0.09, p = 0.41) indicating an effect of fatty liver on renal function. To address renal function, we analyzed the eGFR which was inversely correlated with RSF (Supplementary Fig. S8), suggesting lower eGFR for subjects with higher RSF (r = 0.24, p < 0.0001). However, we found no association between fetuin-A levels and RSF (p = 0.76 after adjustment for sex, age, BMI) but a trend for a positive association between fetuin-A levels and BMI (estimate = 1.5, p = 0.06).

## Discussion

Previous investigations have indicated that fat cells from different anatomical sites are different in morphology and in their cytokine and adipokine expression pattern^7, 24^. In the present study, we investigated the cytokine expression of RSF from human donors and its effect on human glomerular EC and PO. We strictly remained in the human system, although there are disadvantages when compared to animal studies, e.g. the number of donors is limited, the donors are less well characterized and heterogeneous.

First, we characterized this unique perivascular fat compartment: we compared RSFC with PVFC, but also with VFC and SCFC. We found that (i) RSFC and PVFC are similar in morphology and differentiation capacity, but differ from VFC and SCFC. ii) RSFC show a similar high expression of (pro)-inflammatory factors compared to VFC, and even the highest expression of the chemoattractant MCP-1 and the regenerative factor HGF. Our data are well in line with earlier reports showing that fat depots from different anatomical locations differ in many aspects^7, 24^. However, the characteristics and functional influence of RSFC on kidney cells had not been studied. In cocultures with EC and PO, RSFC exerted beneficial effects on glomerular cells by downregulation of (pro)-inflammatory and chemoattrative factors and by strongly increasing HGF mRNA-expression and protein secretion in EC. This indicates a proregenerative and antifibrotic effect of healthy RSF, particularly since the the profibrotic antagonist TGF-β was reduced (Fig. 5). Our results indicate that RSF and glomerular cells are active and regulated producers of factors, which may influence kidney function via secreting factors, rather than compressing the renal artery by the augmentation of this ectopic fat compartment, as suggested previously^16, 18, 25^.

Recent data have pointed to an impact of the fatty liver on renal function^21^. Our present work complements this finding by showing that signals from the fatty liver adversely modulate the renal impact of RSF (Fig. 5). Therefore, we also examined the influence of fatty liver derived fetuin-A ± palmitate on RSFC. Similar to PVFC^14^, treatment of RSFC with fetuin-A/palmitate caused a substantial increase in the expression of (pro)-inflammatory factors, indicating a potentiation of the (pro)-inflammatory status, while HGF was inhibited. In RSFC we found that downstream of TLR4 activation by fetuin-A/palmitate the NFκB and JNK signalling pathways are involved. Consequently, the regenerative effects of RSFC-derived HGF on EC and PO were reduced. Thereby the benign effects of HGF, e.g. the preservation of PO structure and anti-apoptosis^26^ may be negatively affected. Thus, fetuin-A/palmitate - via the alteration of HGF secretion in RSFC - may impair glomerular barrier function. The involvement of different signalling pathways may explain the varying effects of fetuin-A on (pro)-inflammatory versus regenerative factors, particularly HGF.

The fetuin-A treatment of RSFC cocultured with EC and PO turned the reduced (pro)-inflammatory state of glomerular cells into an induced (pro)-inflammatory state (Fig. 5). Furthermore, these conditions stimulated the expression of the chemoattractant MCP-1 and a trend for stimulation of the adhesion molecules ICAM-1 and ALCAM in RSFC, EC and PO was observed probably leading to the attraction and invasion of leukocytes, particularly monocytes, to the glomerulum (Fig. 5). Indeed, monocyte-derived macrophages are found in the glomerulum in obesity-related kidney disease^27^. Similarly, fetuin-A caused also an increased expression of the chemoattractant MCP-1 in RSFC. Accordingly, we detected M1/M2 macrophages in human renal sinus fat resections. In addition to fat cells, these adipose tissue macrophages can produce large amounts of TNF-*α*, IL-1*β*, IL-6 and MCP-1^28^. Since no immune cells, like monocytes/macrophages, were present in our isolated primary cells (fat tissue *in vivo* contains fat, endothelial and immune cells), other important cytokines, such as TNF-α and IL-10, were not expressed per se by RSFC, EC and PO unless stimulated by immune cells. In our ongoing coculture studies, including monocytes, those cytokines will be also examined. Noteworthy, the selective inhibition of MCP-1 has been proven to reduce macrophage accumulation and renal injury^29–31^. The increased HGF production by EC may act in a paracrine fashion on adjacent PO, that in turn lack HGF expression, as shown here by our group and others^32^. Given that PO express the corresponding c-met receptor, they may respond to EC-derived HGF. Administration of HGF was previously reported to improve proteinuric kidney disease, because it preserves PO structure^33^. However, the fetuin-A-induced elevation of (pro)-inflammatory factors and TGF-*β*, and the slight HGF-downregulation in glomerular cells may enhance vascular permeability and reduce glomerular barrier function^34^. The strong induction of (pro)-inflammatory factors may outweigh, or even overwhelm, HGF effects on barrier function. HGF acts as an antifibrotic factor which antagonizes the effects of TGF-*β* ^35^. Thus, the balance between the oponents HGF and TGF-*β* is also altered, which may influence kidney morphology and pathophysiology, possibly contributing to obesity-related nephropathy (Fig. 5). Furthermore, we found a slight inhibition of CTGF which is a major downstream profibrotic factor of TGF-β. CTGF have been proposed to be involved in podocyte injury. Podocyte injury and loss are found in the very early stages of diabetic nephropathy^36^.

Previous studies have demonstrated the presence of interindividually varying amounts of fat around the vessels of the renal hilum in humans^15–17^ potentially influencing renal/glomerular function via organ crosstalk. Accordingly, in a large subcohort of the Framingham study, quantification of RSF accumulation (area measured planimetrically in representative CT images) was independently associated with both hypertension and chronic kidney disease (CKD)^15, 37^. Our study was performed in 449 healthy subjects at increased risk to develop T2D. We found that RSF was associated with albuminuria in subjects with fatty liver. In this subgroup, albuminuria also associated with increased fetuin-A levels. In addition, RSF inversely correlated with eGFR. This indicates that RSF, particularly in conjunction with fatty liver, may influence renal function in apparently healthy individuals. Recently, a study was published analysing a population-based cohort comprising 37,496 participants. Strikingly, 20.1% of participants with NAFLD had impaired renal function compared to only 8.7% in those without NAFLD^38^. These new data suggest again that there is an association between NAFLD and impaired renal function.

Our results together with previous reports indicate liver-kidney interactions in NAFLD^39^. How can patients profit from these new insights? Since both the kidney and the liver express all components of the renin-angiotensin-system, an intervention may address both pathologies. Accordingly administration of the angiotensin receptor blocker valsartan decreased both fetuin-A levels and uACR^40^. Noteworthy, also in an animal model elevated fetuin-A levels released by the fatty liver were found to be associated with microalbuminuria and diet-induced hepatic steatosis could be improved by treatment with fenofibrate. Both elevated fetuin-A levels and albuminuria were reduced^41^. Similarly, treatment of obese patients with fenofibrate decreased fetuin-A levels; unfortunately uACR was not analysed^42^. Together these results indicate that the pathologies of both organs may be hit by application of one drug.

To the best of our knowledge, the interplay between RSF and glomerular cells from human origin via paracrine factors has not been studied yet. Also *in vitro*, we strictly remained in the human setting because we had the unique opportunity to obtain primary cells from human allografts before transplantation. Our results indicate that in a metabolically benign condition RSF reduces the release of (pro)-inflammatory factors by EC and PO. These findings, together with previous reports showing that perivascular tissue is a modulator of vascular tone by adipocyte-derived relaxing factor indicates a benign effect of perivascular fat tissue^43, 44^. However, it was also shown that PVAT displays anti-contractile properties involving (ADRF), a transferable substance with direct vasodilator effects^45^. However, in a metabolically malignant condition, when fatty liver-derived hepatokines, like fetuin-A, act on human RSF, the beneficial influence of RSF on glomerular cells is abolished, possibly leading to renal dysfunction and damage.

## Methods

### Cell culture and treatment

Preadipocytes were isolated from abdominal subcutaneous, visceral, perivascular and renal sinus fat resections of each of 5 patients (age 56–67 years) who underwent abdominal, hand, coronary bypass or renal surgeries for clinical purposes and gave informed written consent. We confirm that all methods were performed in accordance with the relevant guidelines and regulations described in the proposal submitted to the Ethical Committee. These procedures were finally approved by the Ethical Committee of the University of Tübingen (UKT No. 300/2012BO1 and BG-Clinic Nr. 205/2011BO2). Isolation, cultivation, differentiation and oil red staining of fat cells (Supplementary Methods) were performed as previously described^7^ and characterized by fluorescence-activated cell scanning with specific markers (Supplementary Fig. S1). Human arterial endothelial cells (EC) were isolated from healthy radial artery specimens which were discarded after bypass surgery^7^. Finally, a well-characterized conditionally immortalized human podocyte (PO) cell line was used which was kindly provided by Prof. Saleem^46^. Cocultures were performed as previously described^7^. Parts were also (pre)treated with 600 µg/ml fetuin-A (alpha-2-HS-glycoprotein, Sigma, endotoxin <0.05 EU/µg or 0.005 ng/µg determined by ELISA Kit Hycult Biotech) ± 50 µM palmitic acid in BSA (Sigma) and several inhibitors (Supplementary Methods).

### Quantification of protein levels

HGF concentrations were measured by sandwich enzyme immunoassay technique (R&D Systems, Wiesbaden-Nordenstadt, Germany). The human IL-6, IL-8 and MCP-1 ELISA (MAX™ Deluxe from BioLegend Europe BV Uithoorn, The Netherlands), and VEGF ELISA (IBL International distributed by Tecan, Switzerland) were performed according to the manufacturer’s instructions. Furthermore, the Luminex xMAP® technology (Luminex Corporation, Austin, TX USA) was used.

### Real-time PCR analysis

A real-time PCR analysis was used to quantify the transcripts of the proteins of interest (Supplementary Methods), as previously described^7^.

### Study participants

We studied a total of 449 participants of the Tuebingen Family Study (TUEF) which is an ongoing cross-sectional investigation aimed to metabolically characterize a population at increased risk for T2D. Subjects with a family history of T2D, prior gestational diabetes, known glucose intolerance or overweight were invited to take part in a metabolic assessment involving an oral glucose tolerance test. The subjects were considered healthy according to a medical history, physical examination and routine laboratory tests. They had no history of liver disease and did not consume more than two alcoholic drinks per day. Body fat mass and distribution, including the amount of RSF, and liver fat content were measured in a subgroup using whole-body magnetic resonance (MR) imaging and liver spectroscopy. No individual study participant can be identified by the values shown in the Table 1 and in the MR images. The anthropometric and clinical data of the studied cohort is shown in the Table 1. The ethics committee of the Medical Faculty of the University of Tübingen approved the study protocol (UKT No. 422/2002). All methods were performed in accordance with the relevant guidelines and regulations. The participants were interviewed face-to-face and a written, informed consent was obtained from all participating individuals.

### Assessment of renal sinus fat and intrahepatic lipid content

Body fat mass and distribution, liver fat content^47^ and the amount of RSF^17^ were measured as previously described. Subjects with a liver fat content >5.6% were considered to have NAFL^47^. For RSF, the area of renal tissue and the area of adipose tissue in the renal sinus were measured planimetrically on the level of the renal hilum in transverse magnetic resonance images, separately on both sides. Based on these tissue areas a mass indicator of RSF (RSF_MI) was derived for each subject. RSF_MI and liver fat content (assessed by MR-spectroscopy) were determined and analyzed to answer the question if RSF interacts with liver fat (p = 0.03).

### Clinical laboratory measurements

Plasma and urine creatinine were measured enzymatically by the creatinkinase method and albuminuria was measured nephelometrically in 430 subjects, as described^17^. Participants with positive urinary leukocyte esterase strip tests (n = 75) were excluded from the albuminuria studies.

### Immunohistochemical stainings

HE and immunostainings were performed at paraffin sections by the Institute of Pathology Tübingen with automated protocols using the Opti-View Kit (Roche-Ventana, Basel, Switzerland) and antibodies against CD68, CD206 and CD31 (Supplementary Methods).

### Presentation of data and statistics

For the *in vitro* data, statistical methods are indicated in detail in the respective figure legend. These analyses were done using R version 3.3.3 and Graph PadPrism (GraphPad, La Jolla, CA, USA). The data are expressed as mean ± SEM. For all analyses, *p*-values < 0.05 were considered statistically significant. Random-slopes linear mixed models were conducted for time-repeated measurements, adjusting for the donors and the observation time as random effects. Furthermore, differences between means were tested with ANOVA, for post-hoc testing of contrasts Tukey’s “honest significant differences tests” were performed. For the analysis of differences in measurement pairs from the same donor, paired t-test was conducted.

The estimated glomerular filtration rate (eGFR) was calculated by the CKD-EPI equation that is, in contrast to other commonly used formula, more suitable to assess eGFR in a healthy population^48, 49^. Linear regression analyses were performed with the least-squares method. Variables with skewed distributions such as uACR and RSF were appropriately transformed prior to analyses. RSF was adjusted for sex, age and BMI, when not otherwise noted. The effect size of the independent variable is given as standardized beta. For these calculations, JMP 11 (SAS, Cary, NC) was used.

## Electronic supplementary material

Supplementary information
