# Supplementary material for: The protective effect of human renal sinus fat on glomerular cells is reversed by the hepatokine fetuin-A
Source: Sci Rep. 2017 May 23;7:2261. doi: 10.1038/s41598-017-02210-4 (PMC5442123; doi:10.1038/s41598-017-02210-4)
Supplement: Supplementary file 1 — Supplementary information [file 41598_2017_2210_MOESM1_ESM.pdf]

The protective effect of human renal sinus fat on glomerular cells is reversed by the hepatokine fetuin-A

R. Wagner[1,2,3], J. Machann[2,3,4], M. Guthoff[1], P. Nawroth[3,5], S. Nadalin[6], M.A. Saleem[7], N. Heyne[1], A. Koenigsrainer[6], F. Fend[8], F. Schick[2,3,4], A. Fritsche[1,2,3], N. Stefan[1,2,3], H.-U. Häring[1,2,3], E. Schleicher[1,2,3], *D. I. Siegel-Axel[1,2,3]

1. Department of Internal Medicine IV, Division of Endocrinology, Diabetology, Angiology, Nephrology and Clinical Chemistry, University of Tübingen, Germany
2. Institute for Diabetes Research and Metabolic Diseases of the Helmholtz Center Munich at the University of Tübingen
3. German Center for Diabetes Research (DZD), München-Neuherberg, Germany
4. Section on Experimental Radiology, Department of Diagnostic and Interventional Radiology, University of Tübingen, Germany
5. Department of Endocrinology and Clinical Chemistry, University of Heidelberg, Germany
6. Department of General Visceral and Transplantation Surgery, University of Tübingen, Germany
7. Bristol Royal Hospital for Children, Paediatric Renal Medicine, University of Bristol, UK
8. Institute of Pathology and Neuropathology, University ofTübingen, Germany

***Corresponding author:** Prof. Dr. Dorothea D.I. Siegel-Axel, Department of Internal Medicine, Division of Endocrinology, Diabetology, Angiology, Nephrology, and Clinical Chemistry, Eberhard Karls University Tübingen, Otfried-Müller Str.10, D-72076 Tübingen, Germany.

Phone: +49-7071-2982906. Fax : +49-7071-294460, E-mail: dorothea.axel@med.uni-tuebingen.de

Supplementary Methods

Transwell coculture system

6-well or 24-well dishes with polycarbonate membrane inserts (pore size of 3.0, Greiner, Germany) were used. Primary preadipocytes were seeded on six-well plates at a density $5x10^4$ cells/well or on 24-well plates at a density of $1x10^4$ cells and cultivated for 3 days in monocultures. EC were seeded on the upper side of a gelatine-coated transwell inserts at a density of $1x10^4$ cells/well (24-well plate) or $5x10^4$ cells/well (6-well plate) and also cultivated for 3 days. After reaching confluence, filter membranes covered with EC were inserted into the six-well plates containing confluent RSFC on the bottom of the culture dish. By this construction, two culture compartments were created (PA-compartment and haEC-compartment). The same model was also used for the cocultivation of RSFC with PO. All cell types were cultured in same culture medium (Vasculife ENGS) because previous experiments showed that this medium supports both endothelial and RSFC growth. Thus, a potential medium gradient should be avoided between the two compartments. After 24 h, 48 h and 72 h, conditioned media from each compartment were collected separately and stored at -80°C. Finally, cells were lysed by lysis buffer (Macherey-Nagel, Düren, Germany, +ß-mercaptoethanol 1:100), and stored at -80°C.

Treatment conditions

Parts of mono- and cocultures were (pre)treated with 600 µg/ml fetuin-A (alpha-2-HS-glycoprotein, Sigma, endotoxin <0.05 EU/µg or 0.005 ng/µg determined by ELISA Kit Hycult Biotech) ± 50 µM palmitic acid in BSA (Sigma) and several inhibitors.

Other parts were cultured in the same way but pretreated with the JNK inhibitor SP500125 (10 µmol/L), the p38MAPkinase inhibitor SB203580 (10 µmol/L), the MEK1/2 inhibitor PD98059 (10 µmol/L) and the NFκB inhibitor BAY11-7082 (1 µmol/L) 1 h before the addition of fetuin-A or 50 µM palmitic acid in BSA (Sigma) or Lipopolysaccharide (LPS) from *S. minnesota* R595 (5

ng/ml, Alexis). After 24h conditioned media from each compartment were collected separately and stored at -80°C. Monocultures were performed under exactly the same conditions but lacking the second cell type. Finally, cells were lysed and stored at -80°C, according to the manufacturer's instructions (lysis buffer +ß-mercaptoethanol 1:100, Macherey-Nagel, Düren, Germany).

Measurement of mRNA expression and primer

RNA expression was measured by real-time quantitative PCR on the LightCycler 480 System (Roche) using kits (LightCycler 480 Probes Master; Roche). The mRNA content is given in arbitrary units. The mRNA-expression of all studied proteins was normalized using the *RPS13* housekeeping gene product as an endogenous reference. The relative gene expressions (e.g. cocultures versus monocultures or Fet-A treated cells versus untreated cells) were calculated using cycle threshold (CT) values in accordance with the Δ/Δ CT method.

The primers and probes were designed using Universal Probe Library (Roche Applied Science).

Gene-specific probes and primer pairs are the following:

ALCAM for GGAGGAATATGGAATCCAAGG, rev CTGAATTTACAGTATACCATCCAAGG

ICAM GCTGGAGCTGTTTGAGAACAC, rev CAAGTTGTGGGGGAGTCG

IL-6 for AGCTATGAACTCCTTCTCCACAA, rev GGTACTGGGGCAGGGAAG,

IL-8 for AGACAGCAGAGCACACAAGC, rev AGGAAGGCTGCCAAGAGAG,

MCP-1 for CTGCTCATAGCAGCCACCTT, rev GCACTGAGATCTTCCTATTGGTG,

HGF for GCATGTCCTCCTGCATCTC, rev TTCTTCTTTTCCTTTGTCCCTCT,

VEGF-A for CTACCTCCACCATGCCAAGT, rev CCATGAACTTCACCACTTCGT,

RPS13 for CCCCACTTGGTTGAAGTTGA, rev ACACCATGTGAATCTCTCAGGA

The amplification conditions were 5 min at 95°C, a cycle of 95°C for 10 sec, and 60°C for 30 sec and 72°C for 1 sec, for a total of 40 cycles. RNA was extracted using a kit (Nucleo Spin RNAII, Macherey-Nagel, Düren, Germany) according to the manufacturer's instructions. For reverse transcription PCR, 1 µg of RNA was used with random hexamer primers and a cDNA synthesis

kit (Transcriptor First Strand; Roche Diagnostics Deutschland GmbH, Mannheim, Germany) according to the manufacturer's instructions.

Immunohistochemical procedure

Immunohistochemical stainings of 4-μm-thick paraffin sections were performed using the Opti-View Kit (Roche-Ventana, Multimer technology) which is a hapten-multimer system without biotin which minimizes background stainng. The following primary antibodies were used: monoclonal mouse anti-human CD31 (1:100, clone JC70A, Dako Glostrup, Denmark), ployclonal rabbit anti-human CD206 (1:8,000, clone ab64693, Abcam, Cambridge, Massachusetts) and monoclonal mouse anti-human CD68 (1:3,000, clone KP-1, Dako).

Supplementary figures

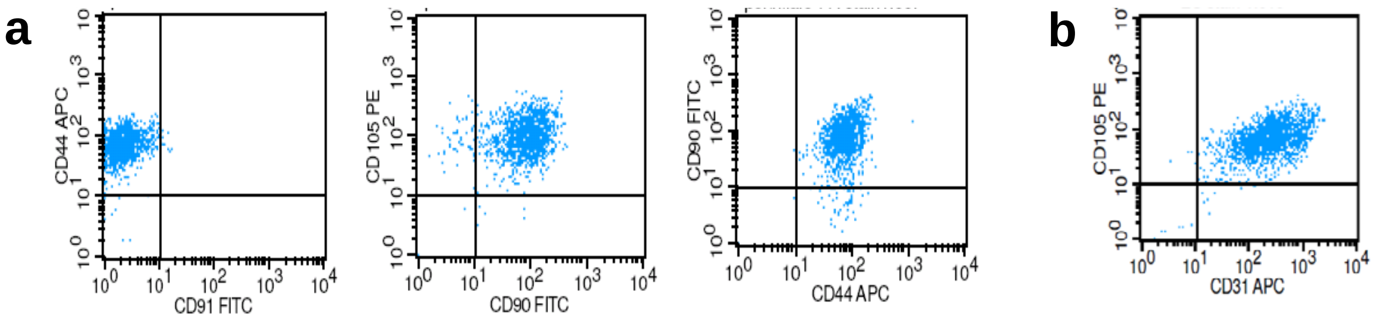

Figure S1: FACS analysis: double labeling of **(a)** RSFC **(b)** and EC with antibodies against the adipocyte stem cell marker marker CD90, CD44, and CD105, and the EC-specific CD31 antigen.

Supplementary figures

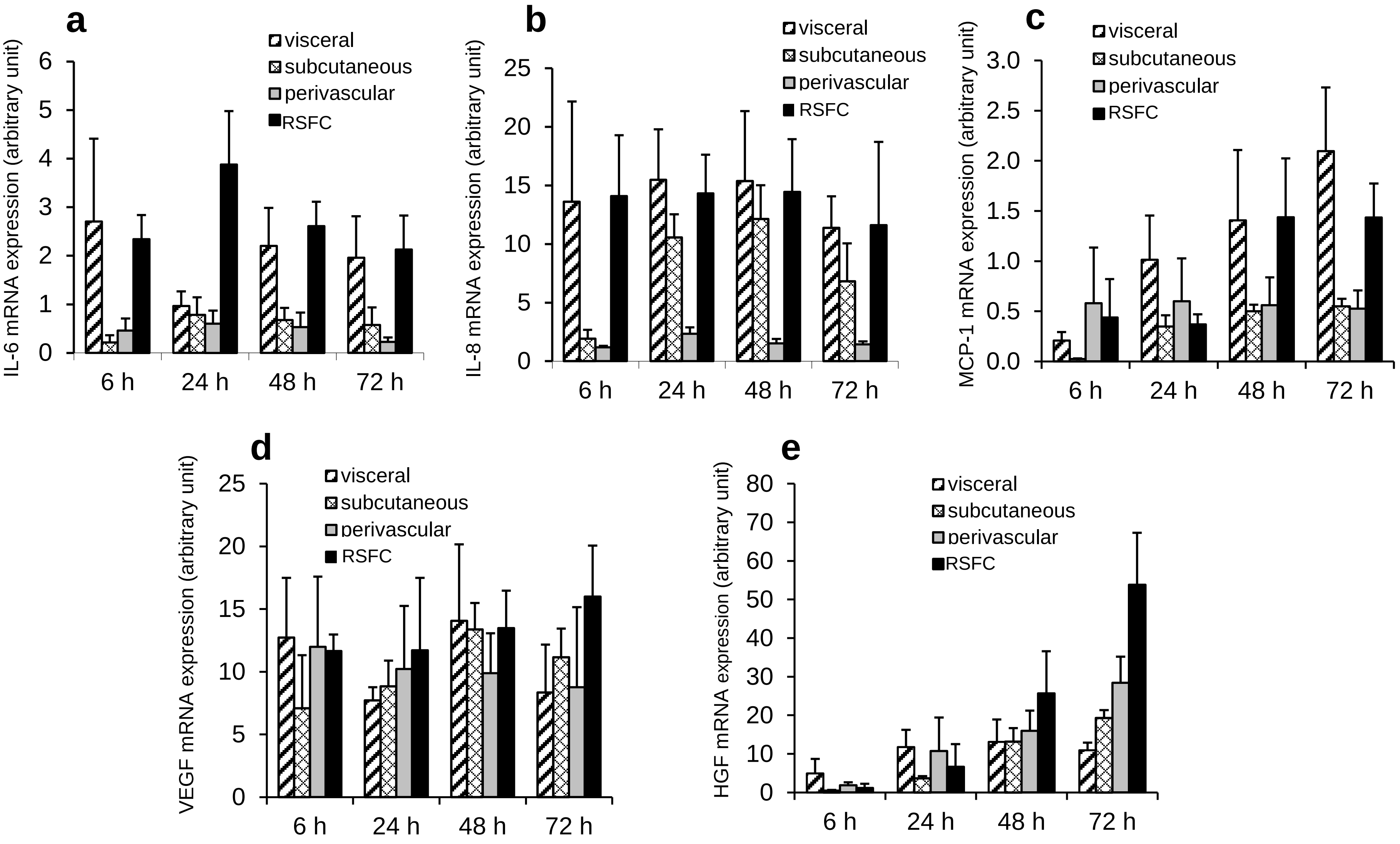

Figure S2: Comparison of the time dependent mRNA-expression of a variety of different angiogenic and pro-inflammatory proteins in visceral, subcutaneous, perivascular and RSFCdetermined by real-time PCR (light cycler) after 6, 24, 48 and 24 h cultivation. **(a)** IL-6, **(b)** IL-8, **(c)** MCP-1, **(d)** VEGF and **(e)** HGF. Values are mean SEM from four different experiments (basal mRNA expression of each protein versus the housekeeping gene RPS13).

Supplementary figures

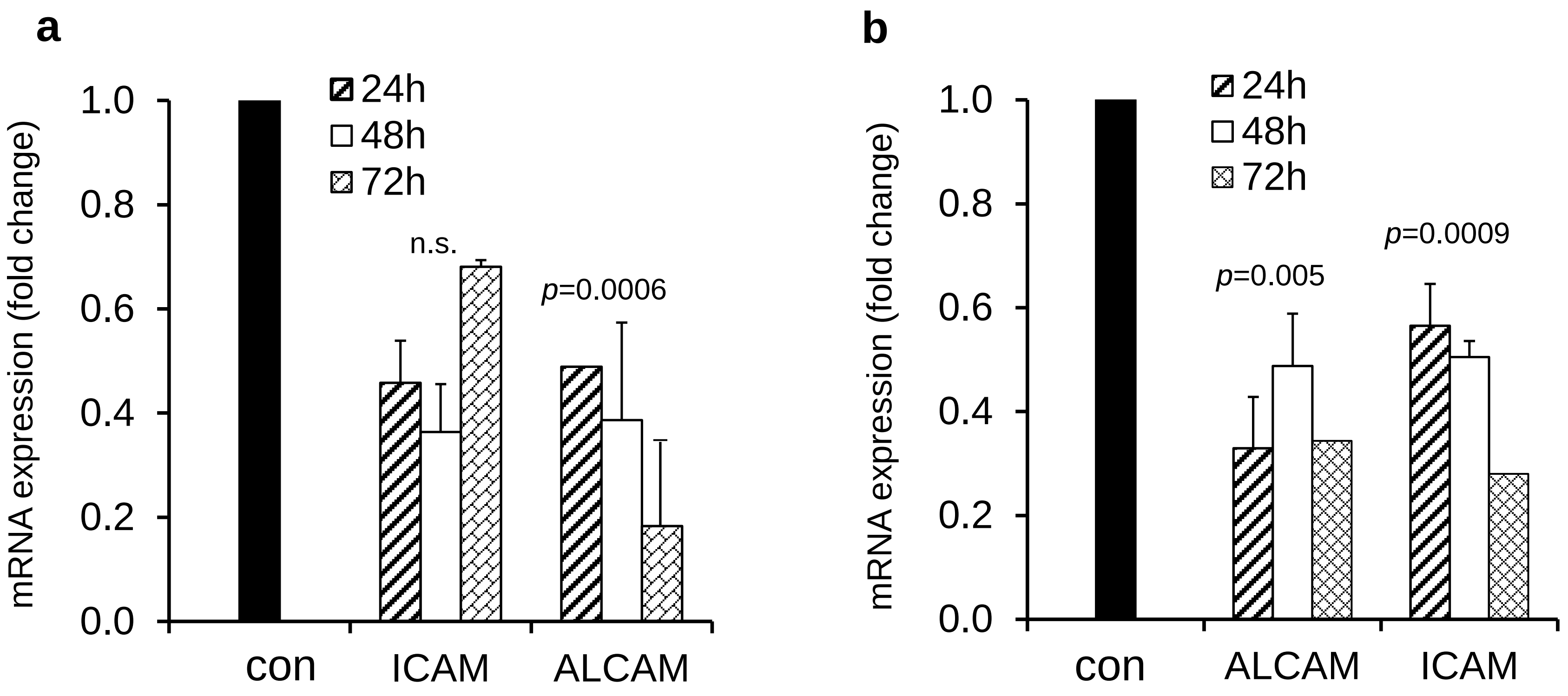

Figure S3: Stimulation or inhibition of the mRNA expression of the adhesion molecules ICAM-1 and ALCAM in cocultivated **(a)** EC and **(b)** PO. Quantitative mRNA-expression determined by real-time PCR (light cycler) in lysed cells after cocultivation for 24, 48 and 72h using the transwell system. Values are mean SEM from 3 experiments. Mixed model with random slopes, *p* for difference of all time-points compared to controls; *p*-values are shown for cocultures versus monocultures (con) defined as 1 (black bars), *p*-values <0.05 were considered statistically significant.

Supplementary figures

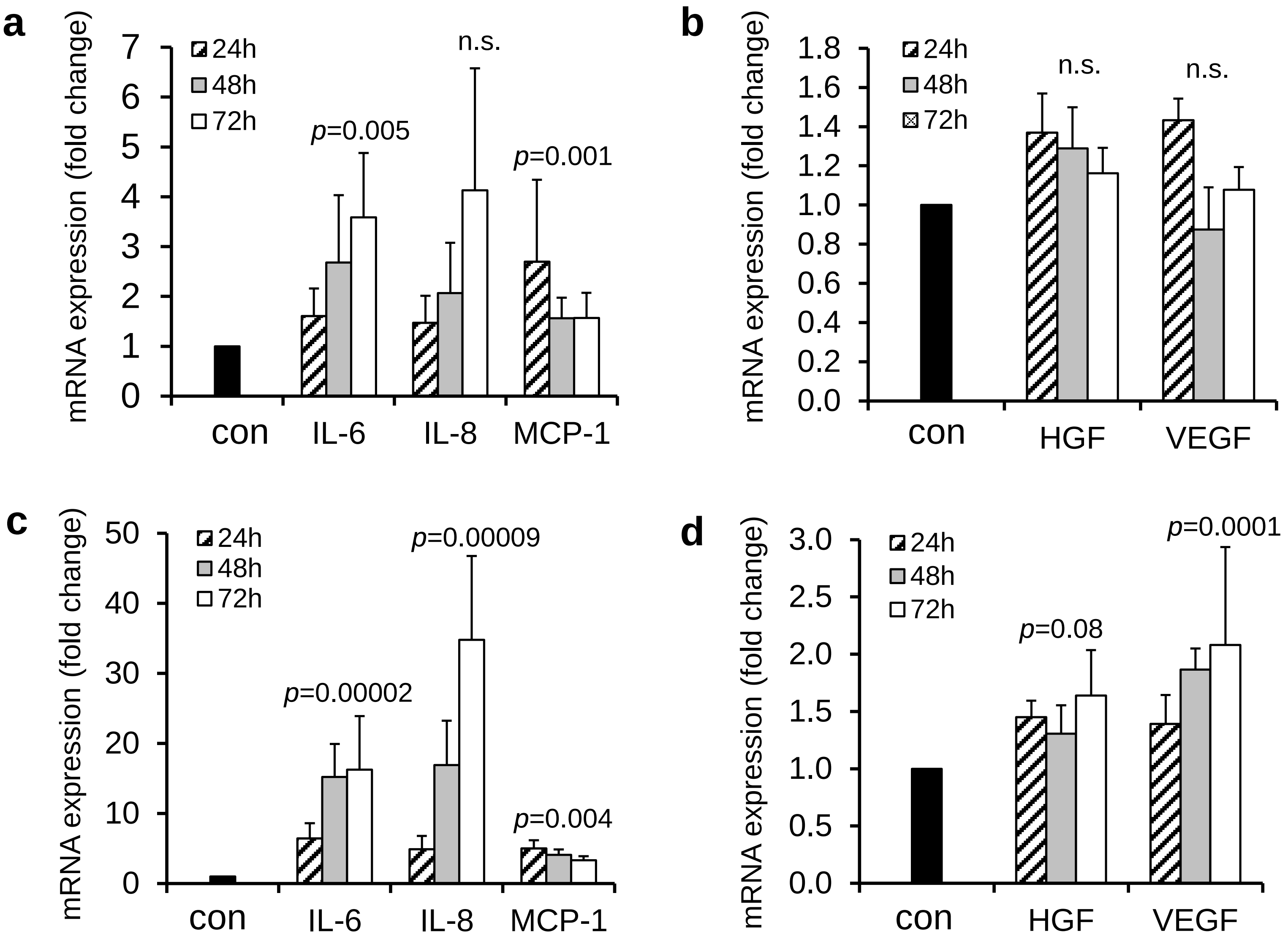

Figure S4: Stimulation or inhibition of the mRNA expression in RSFC cocultivated with **(a, b)** EC or **(c, d)** PO. Quantitative mRNA-expression of proinflammatory (left panels) and angiogenic factors (right panels) determined by real-time PCR (light cycler) after cocultivation for 24, 48 and 72h in the transwell system. Values are mean SEM from 6 experiments (mixed model with random-slopes, *p* for difference of all time-points compared to controls; *p*-values are shown for cocultures versus monocultures (con) defined as 1 (black bars), *p*-values <0.05 were considered statistically significant.

Supplementary figures

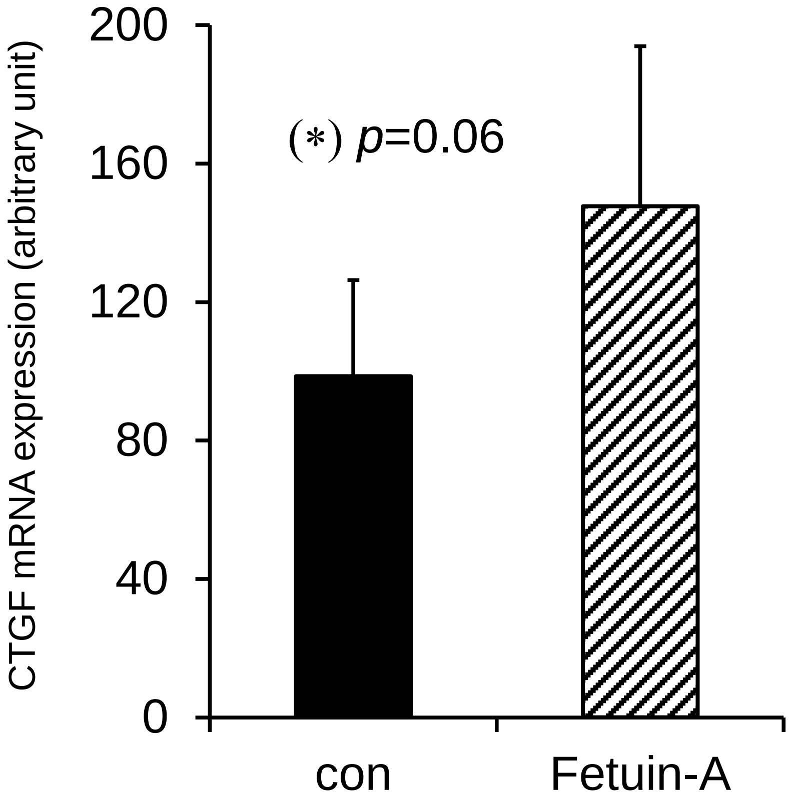

Figure S5: Quantitative mRNA-expression of CTGF determined by real-time PCR (light cycler) in lysed cells after cultivation with or without 600 µg/ml Fet-A for 24h. Basal mRNA expression of CTGF versus the housekeeping gene RPS13. Values are mean SEM from 4 experiments (paired t-test).

Supplementary figures

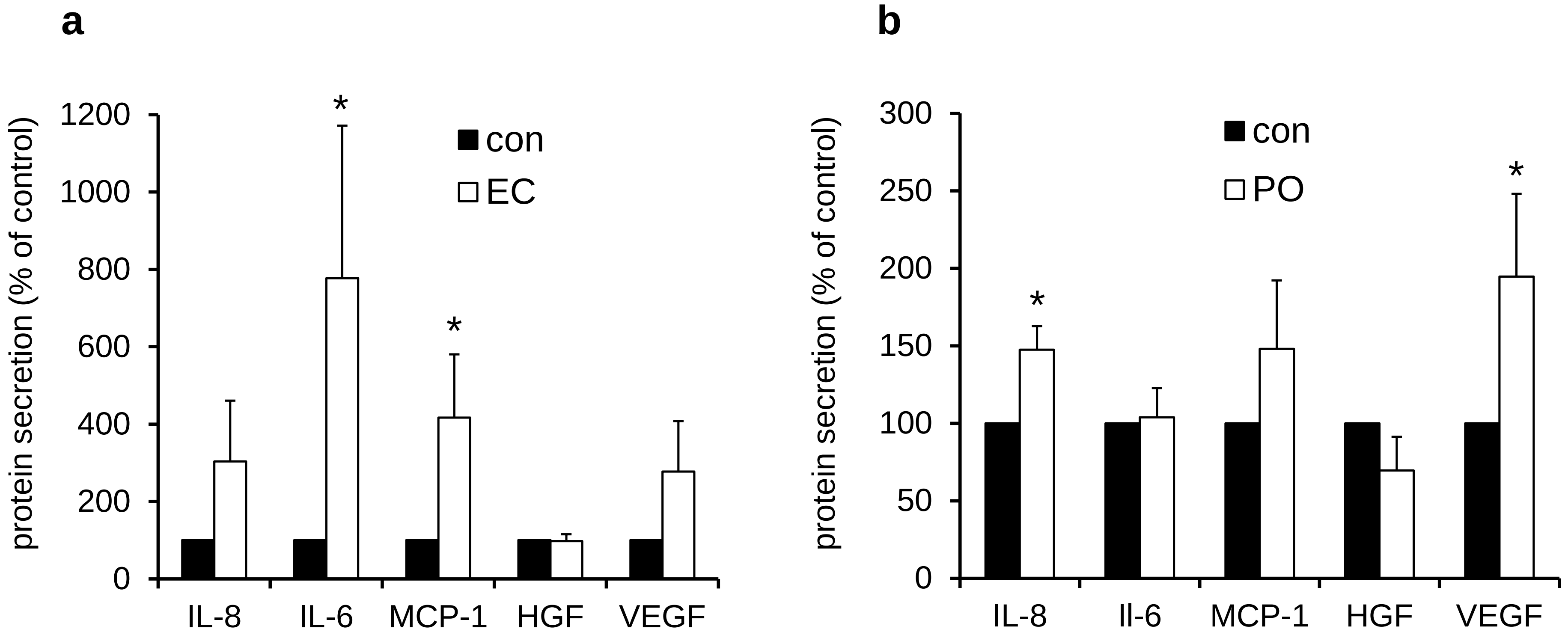

Figure S6: Effects of Fet-A on the secretion of representative proinflammatory and angiogenic proteins. Percental protein levels in supernatants of **(a)** EC and **(b)** PO after cocultivation with RSFC for 48h (untreated controls, black bars) and after treatment with Fet-A (600 µg/ml, white bars). Values are mean SEM from 4 experiments; paired t-test, *p*-values are shown for cocultures versus monocultures (con) defined as 1 (black bars), *$p<0.05$ was considered statistically significant.

Supplementary figures

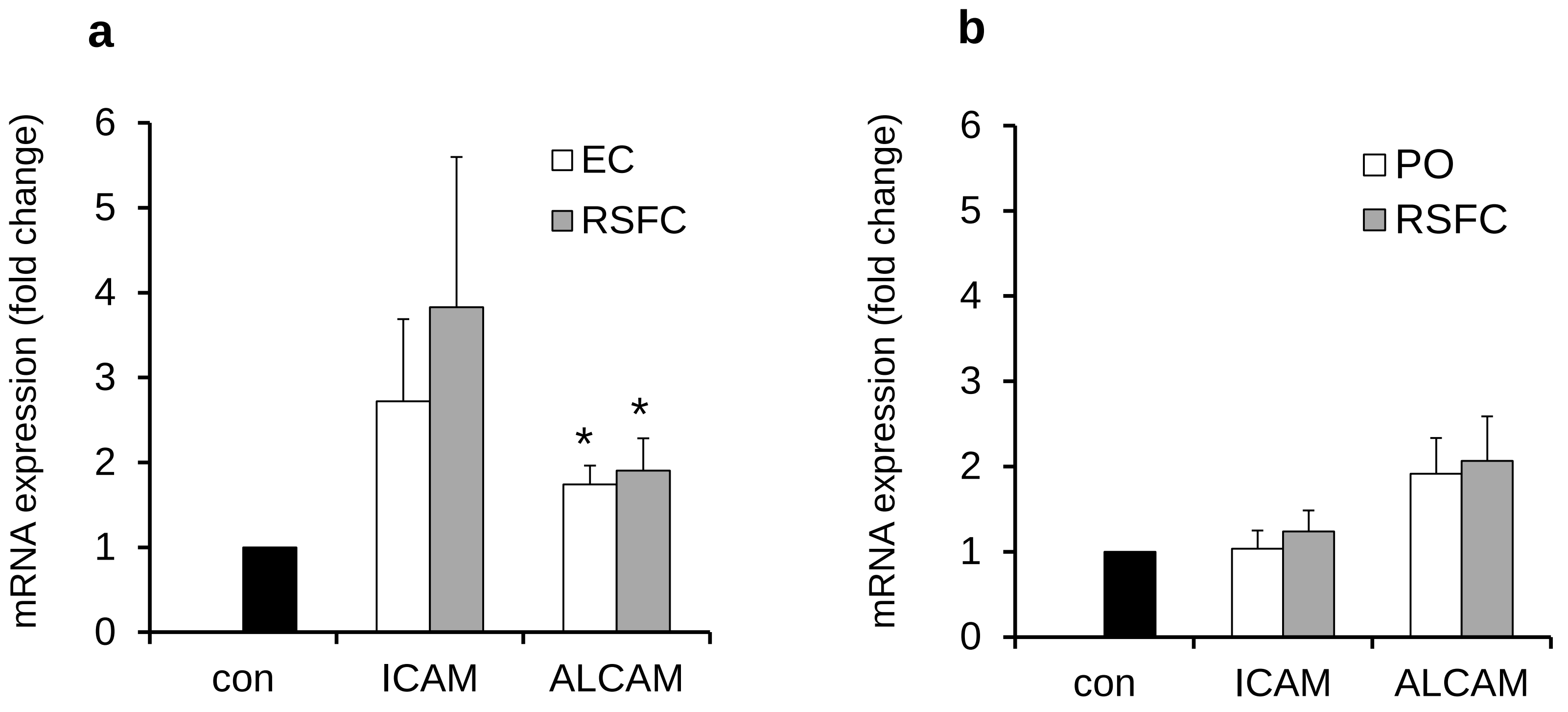

Figure S7: Effects of fetuin-A on the mRNA expression of the two adhesion molecules ICAM-1 and ALCAM in **(a)** RSFC cocultivated with EC and **(b)** RSFC cocultivated with PO. Treatment with fetuin-A (600 µg/ml) for 24h. mRNA-expression was determined by real-time PCR (light cycler). Values are mean SEM from 4 experiments. Paired t-test; *p*-values are shown for cocultures versus monocultures (con) defined as 1 (black bars), *$p<0.05$ was considered statistically significant.

Supplementary figures

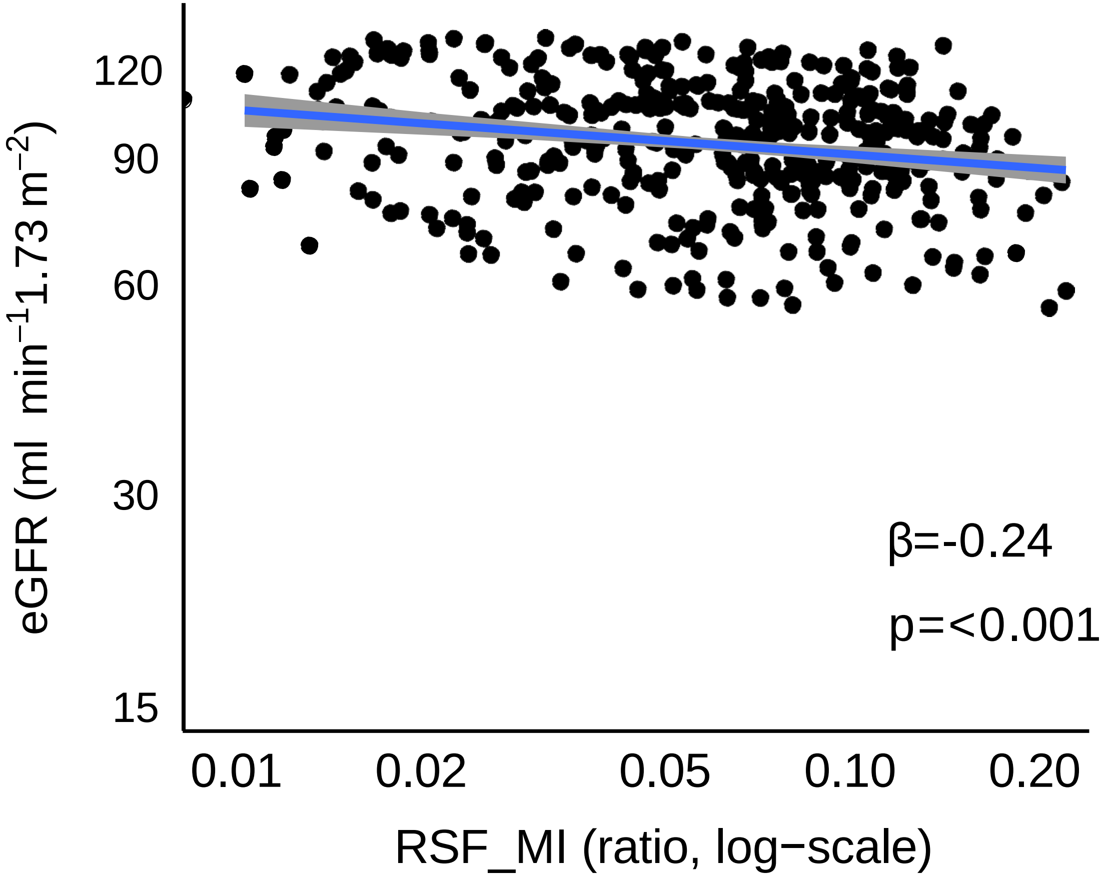

Figure S8: Association of eGFR with RSF_MI. The eGFR was calculated by the CKD-EPI equation. The area of renal tissue and the area of adipose tissue in the renal sinus were measured planimetrically on the level of the renal hilum in transverse magnetic resonance images. 449 residuals adjusted for sex, age and BMI are plotted.
